# Neonatal Testicular Torsion with Hydrocele: A Case Report Underscoring the Need for Early Recognition and Management

**DOI:** 10.1155/2023/9979543

**Published:** 2023-12-11

**Authors:** Kareem Omran, Sameh Ali Ali, Ehsan Ahmad, Hilal Matta, Wissam Jamal Al Tamr

**Affiliations:** ^1^Department of Public Health and Primary Care, University of Cambridge, Cambridge CB2 1TN, UK; ^2^NMC Royal Hospital, Sharjah, UAE; ^3^Surgery Department, Tawam Hospital, UAE

## Abstract

Neonatal testicular torsion (NTT) is a rare but significant condition occurring within the first 30-day postbirth, leading to vascular compromise and potential testicular loss. This paper presents a case of NTT detected incidentally in a neonate with bilateral hydrocele, emphasizing the challenge of early diagnosis and management complexities. The infant underwent surgical intervention involving detorsion and bilateral orchiopexy but eventually required right orchiectomy due to necrosis. The paper highlights the prevalence of NTT in conjunction with hydrocele and stresses the importance of parent education and vigilant follow-up. Various diagnostic methods, primarily ultrasonography, and a range of management strategies are discussed, considering factors such as salvage potential, risk to the contralateral testicle, and surgical intervention's risks and benefits. The paper argues for individualized management, taking into account specific neonate conditions and parental preferences, underlining the essential role of informed and empathetic consultation. The case reinforces the urgent need for increased awareness, early detection, and carefully considered therapeutic approaches to prevent devastating outcomes like infertility and the necessity for lifelong hormone supplementation.

## 1. Introduction

Neonatal testicular torsion (NTT)—a condition manifesting within the initial 30-day postbirth—is a relatively rare but potentially grave paediatric occurrence [1]. It primarily leads to vascular compromise, triggering testicular ischemia and injury. If left unaddressed, this condition may progress to necrosis, ultimately resulting in testicular loss. The optimal approach to managing neonatal testicular torsion remains a subject of debate, specifically regarding the efficacy of surgical intervention in rescuing a testis which has already faced torsion and evaluating the consequent risk to the contralateral testicle [2]. The incidence of bilateral torsion can lead to devastating outcomes, rendering the infant anorchic and consequently infertile, necessitating lifelong hormone supplementation [3].

This case report presents the management of a silent neonatal testicular torsion case, discovered incidentally during a routine visit, and underscores its implications in paediatric surgery. It advocates for increased awareness of this condition and emphasizes the importance of educating parents about potential red flags to enable early detection.

## 2. Case Presentation

**Figure 1:**
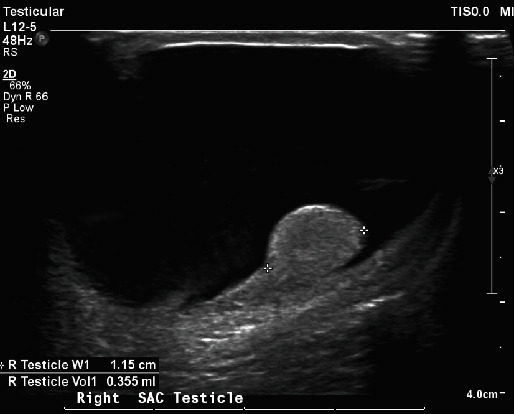
Ultrasound at birth showing large fluid accumulation around a normal right testis.

A male infant born at 40 + 5 weeks through an emergency lower segment caesarean section (LSCS) was observed to have bilateral hydrocele at birth, with otherwise good health, including a high Apgar score and normal feeding behaviour. Initial ultrasonography revealed normal-sized testes with no epididymal abnormalities but significant fluid accumulation in the scrotal sac, suggesting bilateral hydroceles: 25.1 ml on the right side and 22.8 ml on the left (Figure 1).

**Figure 2:**
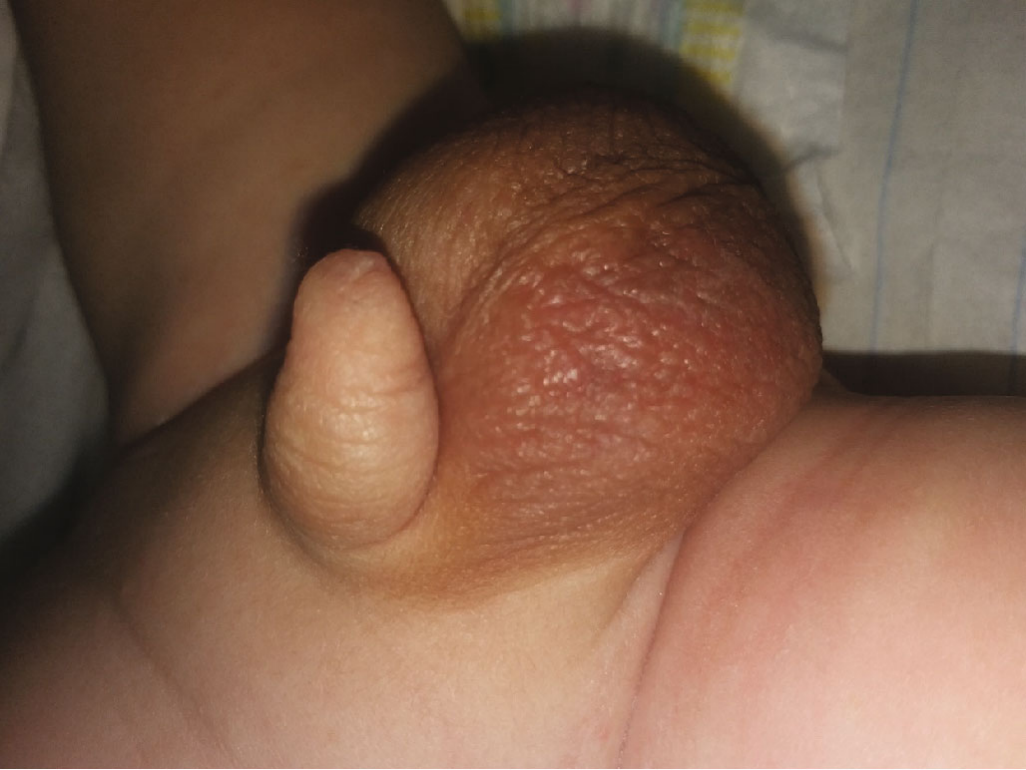
Inflamed and discoloured scrotal sac observed on routine 3-week follow-up visit.

At the 3-week circumcision visit, the physician examined the patient once more and noted a blueish yellow discolouration of the scrotal sac, with an enlarged and hard right testis (Figure 2). The mother revealed that she had noted a bluish yellow discolouration of the right testicle that had persisted for roughly five days. Although this could have been a cause for concern, the infant had no symptoms like poor feeding, vomiting, or diarrhoea and was otherwise active, leading the mother to overlook the discolouration.

**Figure 3:**
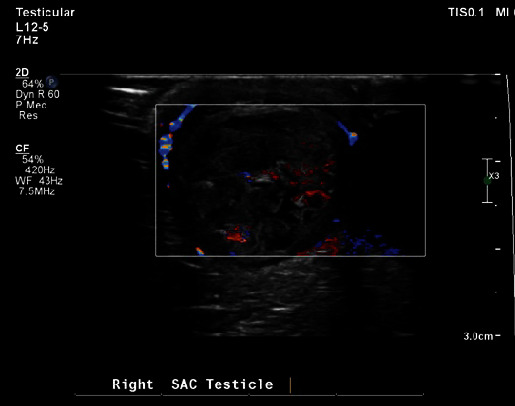
Coloured Doppler ultrasonography showing reduced vascularity in the right testis and increased size.

Ultrasonography at the visit revealed that the right testis had enlarged slightly to measure 0.80 × 0.88 × 1.15 cm (volume 0.43 ml), with a heterogeneous parenchyma echo texture indicating a change in the tissue's structure (Figure 3). The right-sided hydrocele had subsided. Vascularity in the right testis and surrounding tissues was decreased, whereas the left testis appeared normal with a reduced fluid collection of 7.43 ml. These observations suggested a likely torsion in the right spermatic cord/epididymis, a condition known as an intravaginal type of torsion.

**Figure 4:**
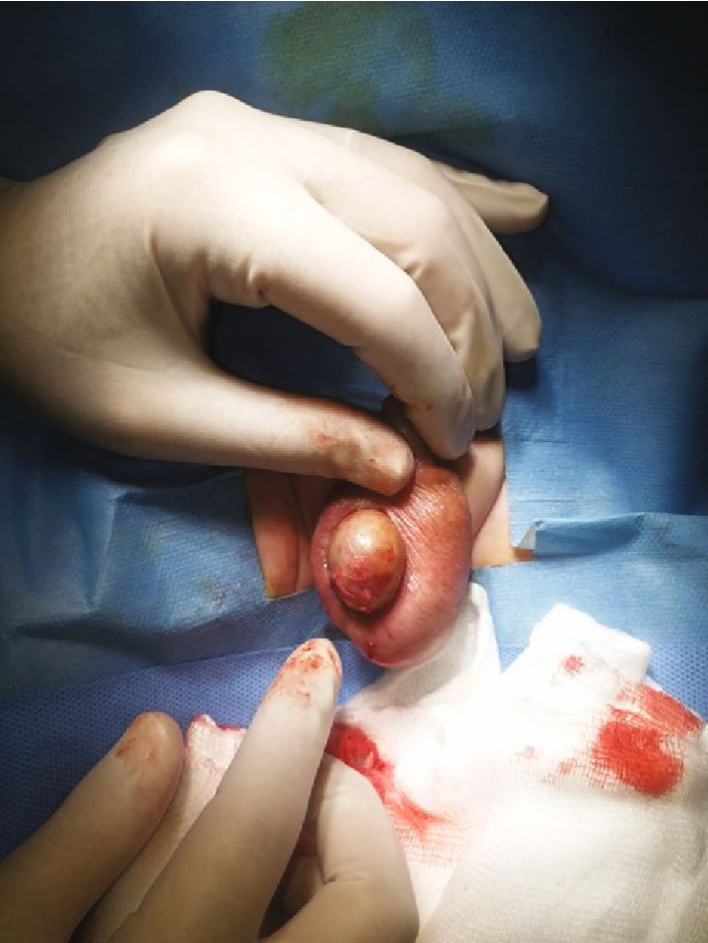
Brown discoloured right testis discovered during the detorsion of the testes and the bilateral orchiopexy procedure.

**Figure 5:**
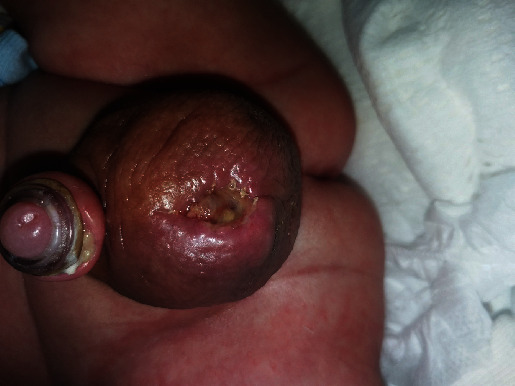
Yellow secretions from the right scrotal sac, suggestive of inflammation and necrosis following torsion of the right testis.

**Figure 6:**
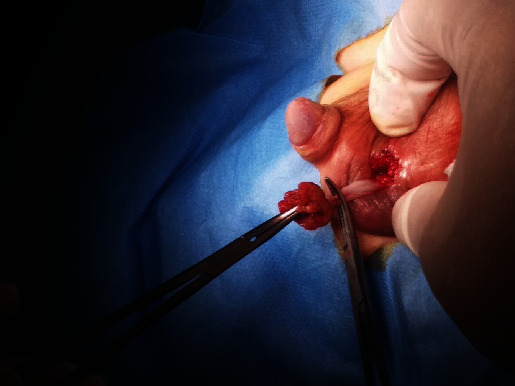
Orchiectomy of the right testis due to a lack of vascularity and necrosis secondary to torsion.

**Figure 7:**
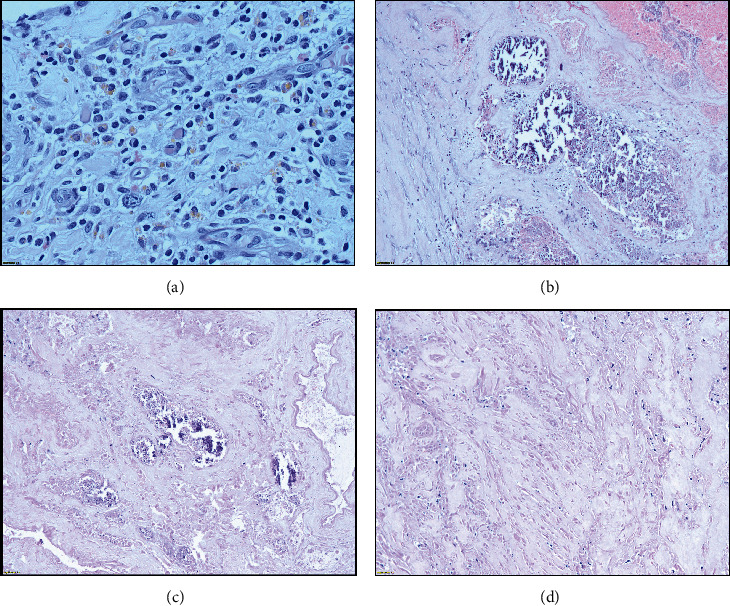
Microphotographs from the necrosed right testis: (a) medium power view (20x) of chronic granulation tissue with old haemorrhage, (b, c) low power (10×) image showing intravascular calcification, and (d) complete infarction of tissue without viable testicular parenchyma.

Following the diagnosis of right testicular torsion, the infant was taken for surgical intervention which included detorsion of the right testicle and bilateral orchiopexy in an attempt to salvage the right gonad. During the operation, it was noted that the right testis was brownish in colour (Figure 4). However, an ultrasound performed a week later (at one month of age) indicated no vascularity in the right testis, though its size and echogenicity appeared normal at 0.9 × 0.9 × 0.9 cm (volume 0.41 ml). Consequently, necrotic tissue and yellowish secretions were observed in the right scrotum (Figure 5), and the patient was taken for a right orchiectomy in surgery (Figure 6). The necrotic remnant of the right testis was then sent for histopathological examination, which showed infarcted vascular spaces with luminal calcifications (Figure 7).

## 3. Discussion

Neonatal testicular torsion (NTT), which may occur prenatally or within the first 30-day postdelivery, is a complex condition characterized by varied clinical presentations and a range of management strategies [4]. It involves testicular ischemia due to vascular compromise, resulting from two distinct pathological processes: extravaginal and intravaginal testicular torsion [5]. Extravaginal torsion, the more prevalent subtype among neonates, encompasses the entire testicle [6, 7]. Concurrently, many affected neonates present with a contralateral hydrocele, which has been identified as a risk factor for NTT [8].

The epidemiological data suggest that NTT has a reported incidence of 6 per 100,000 live births and accounts for 10 to 12 percent of all paediatric testicular torsions [9–11]. In most cases, torsion occurs in utero, representing 70 to 80 percent of cases [4, 9]. Despite a comprehensive understanding of NTT's clinical and pathological aspects, the exact mechanism triggering it is still not entirely understood [12]. Extravaginal torsion is believed to occur after the testicle descends into the scrotum but before it secures itself within it [4, 13]. Some theories suggest that increased intrauterine pressure or complications like breech presentation, preeclampsia, postterm births, difficult vaginal deliveries, and above-average birth weights might predispose the testicle to torsion [9, 14].

Torsion complications primarily involve testicular ischemia, with the severity of damage dependent on the duration of torsion and the degree of spermatic cord rotation [5]. Testicular viability tends to be compromised if ischemia lasts beyond four to six hours [5, 15]. Interestingly, even a less than 360 degrees (partial torsion) can still cause significant damage, although complete torsion (>360 degrees) generally results in greater harm [5, 16]. However, distinguishing between a complete twist and a partial torsion can be a clinical challenge [5]. Regardless, early detection and knowledge of the signs and symptoms are essential for salvaging the gonad. Common symptoms which can be observed and alert towards this diagnosis include a firm or immovable testicle, a “missing” testicle, tenderness, redness, swelling, or firmness of the scrotum—bluish discolouration may also be seen and would be considered a red flag [17]. Differential diagnosis for these presentations of a testicular mass or scrotal swelling in a neonate would include inguinal hernias or neoplasms such as germ cell tumours [18].

Whether an atrophic testicle predisposes the remaining solitary testicle to subsequent torsion remains debatable [19, 20]. Case series have reported conflicting evidence on this issue, with some studies indicating an increased likelihood of subsequent torsion, while others argue the contrary [21, 22]. Limited data suggest that the contralateral testicle can sustain injury independently of concomitant torsion, leading to a recommendation by some experts to remove the torsed testis to protect the contralateral one [23, 24]. However, in bilateral torsion cases, an atrophic testicle may be left in place to preserve some future endocrine function [9, 25].

The diagnosis of neonatal testicular torsion is generally based on characteristic physical findings, which typically include a hardened, fixed, nontender scrotal mass coupled with scrotal discolouration [9]. A definitive diagnosis is confirmed through surgical exploration. In some cases, when the diagnosis remains unclear, diagnostic imaging may be implemented. However, these tests can be challenging to perform in neonates and should not delay emergency intervention if deemed necessary based on clinical examination and presentation [9].

Various diagnostic studies are employed, among which ultrasonography with Doppler colour flow is often the first choice due to its accessibility and noninvasive nature. When operated by an experienced professional, this imaging modality can effectively detect testicular torsion through the absence of detected blood flow to the affected testis [26, 27]. The sensitivity and specificity of ultrasonography with Doppler in diagnosing testicular torsion are quite high, having been reported to range from 69 to 90% and 98 to 100%, respectively [18, 28]. Nonetheless, false-negative and false-positive findings can occur, highlighting the importance of clinical correlation and possibly further diagnostic evaluation or surgical exploration if indicated [29]. Ultrasonography can also reveal other scrotal pathologies on either side of the testis, such as hernias, testicular tumours, or hydroceles [26, 27]. Performing accurate Doppler evaluations in neonates can be challenging, as it can be difficult to detect blood flow in normal neonates, potentially leading to incorrect diagnoses [30]. In our presented case, a significant change was noted in the echogenicity of the testis, suggesting a torsion. This incident brings to attention the importance of routine follow-ups in cases of neonatal hydrocele, as the neonate remained largely asymptomatic.

The management of neonatal testicular torsion is complex and multifaceted, with differing perspectives. The key factors affecting management decisions include the potential to successfully salvage the torsed testis through surgery, the likelihood of subsequent or simultaneous torsion of the contralateral testis, and the risks associated with surgical intervention in the neonatal population, including the need for anaesthesia and/or sedation [1, 2, 4, 9, 31]. A meta-analysis of 197 neonates with testicular torsion found that bilateral scrotal exploration could salvage 7% of torsed testicles and prevent asynchronous torsion in 4% of cases [1]. However, subsequent analysis of 974 infants found that salvage rates were much lower in cases of synchronous bilateral torsion, at 2.5%, compared to 48.5% for asynchronous bilateral torsion events [2]. These findings suggest that approximately 10% of neonates presenting with torsion could potentially benefit from bilateral exploration and orchiopexy of the contralateral testicle [1, 2].

For postnatal torsion, where salvage rates are more favourable at around 30 to 40 percent, emergent surgical intervention is generally advocated, given the potential benefits outweigh the surgical and anaesthetic risks [9, 32–35]. If surgical exploration is conducted, a critical decision is whether to remove a nonviable testicle or leave it in situ. Most experts favour orchiectomy for unilateral torsion due to poor salvage rates, while in bilateral involvement, detorsion and fixation of the testicles are recommended when feasible to preserve potential testosterone production [4, 9, 25]. In the case presented, we chose surgical intervention in an attempt to salvage the affected testicle. However, due to the loss of vascularity, orchiectomy was ultimately performed. This underscores the challenging decision-making involved in managing neonatal testicular torsion.

Another key decision is whether to perform a contralateral orchiopexy, which entails weighing the risk of injury to a healthy, uninvolved testicle against the risk of potential future torsion. This decision also varies globally, with almost all paediatric urologists in the United States performing a contralateral orchiopexy, compared to just 78% of British surgeons due to concerns of injuring the uninvolved testis [35, 36]. For our patient, contralateral orchiopexy was performed during the initial surgery in an attempt to preempt future torsion events. This decision was made considering the relative health of the left testis, as well as the high rate of bilateral torsion, particularly in the context of the hydrocele present in the left testis as well.

On the other hand, some practitioners advocate for nonsurgical management due to overall poor salvage rates and perceived minimal benefit of removing the torsed testicle [37–39]. This approach requires parents to perform serial examinations of the contralateral testicle to ensure that it does not become torsed. While this nonsurgical approach avoids the risks of surgery and anaesthesia, it does carry the risk of potential future torsion of the contralateral testis. Moreover, this approach may require more intensive follow-up and monitoring, which could lead to additional stress for the parents and potential discomfort for the child. In some instances, a conservative approach may be recommended due to certain risk factors, such as prematurity or comorbid medical conditions that make surgery riskier [4, 31]. It is essential to have a thorough and empathetic conversation with parents about the risks, benefits, and uncertainties associated with each treatment approach. The choice of management should be individualized, taking into account the specific circumstances of the neonate, as well as the preferences and concerns of the parents [2, 4, 9, 25, 40]. In the case we have presented, despite the loss of the right testis, the neonate has a good prognosis, particularly with the contralateral orchiopexy that was performed to help safeguard his remaining testicle. Moreover, the possibility of future fertility remains promising, given that the impact of unilateral torsion appears to be minimal [41].
